# *Poria cocos* Regulates Cell Migration and Actin Filament Aggregation in B35 and C6 Cells by Modulating the RhoA, CDC42, and Rho Signaling Pathways

**DOI:** 10.1155/2021/6854860

**Published:** 2021-09-01

**Authors:** Chia-Yu Lee, Chang-Ti Lee, I-Shiang Tzeng, Chan-Yen Kuo, Fu-Ming Tsai, Mao-Liang Chen

**Affiliations:** ^1^Department of Chinese Medicine, Taipei Tzu Chi Hospital, Buddhist Tzu Chi Medical Foundation, New Taipei 231, Taiwan; ^2^Department of Research, Taipei Tzu Chi Hospital, Buddhist Tzu Chi Medical Foundation, New Taipei 231, Taiwan

## Abstract

Poria is used as a traditional Chinese herbal medicine with anti-inflammatory, anticancer, and mood-stabilizing properties. Poria contains triterpenoids and polysaccharides, which are reported to regulate the cytoplasmic free calcium associated with the *N*-methyl-D-aspartate receptor and affect the cell function of neonatal rat nerve cells and hippocampal neurons. Although the modulatory effects of Poria on neuronal function have been widely reported, the molecular mechanism of these effects is unclear. Cell migration ability and the reorganization of actin filaments are important biological functions during neuronal development, and they can be regulated mainly by the Rho signaling pathway. We found that the cell migration ability and actin condensation in B35 cells enhanced by *P. cocos* (a water solution of *P. cocos* cum Radix Pini (PRP) or White Poria (WP)) might be caused by increased RhoA and CDC42 activity and increased expression of downstream ROCK1, p-MLC2, N-WASP, and ARP2/3 in B35 cells. Similar modulations of cell migration ability, actin condensation, and Rho signaling pathway were also observed in the C6 glial cell line, except for the PRP-induced regulation of RhoA and CDC42 activities. Ketamine-induced inhibition of cell migration and actin condensation can be restored by *P. cocos*. In addition, we observed that the increased expression of RhoA and ROCK1 or the decreased expression of CDC42 and N-WASP caused by ketamine in B35 cells could also be restored by *P. cocos*. The results of this study suggest that the regulatory effects of *P. cocos* on cell migration and actin filament aggregation are closely related to the regulation of RhoA, CDC42, and Rho signaling pathways in both B35 and C6 cells. PRP and WP have the potential to restore neuronal cell Rho signaling abnormalities involved in some mental diseases.

## 1. Introduction

*Poria cocos* (Schw.) is a parasitic fungus that exists in various species of *Pinus*. *P. cocos* cum Radix Pini (PRP; Sclerotium Pararadicis, also known as *Fu Shen* in traditional Chinese herbal medicine), and White Poria (WP; also known as *Bai Fu Ling* in traditional Chinese herbal medicine) are medicinal herbs from the dry sclerotium of Polyporaceae fungi that have diuretic, sedative, and tonic effects [1]. They both contain two major types of chemical substances, namely, triterpenoids and polysaccharides. Their other minor ingredients include histidine, amino acids, choline, steroids, and potassium salts [2, 3]. *P. cocos* mediates its pharmacological anti-inflammatory properties via two triterpenoids, namely, pachymic acid and dehydrotumulosic acid [4]. *P. cocos* also has immunomodulatory properties that can alter immune function through dynamically regulated cytokine expression [5, 6]. It has also been reported to possess anticancer properties [2, 7–9]. Moreover, *P. cocos* has been found to regulate the concentration of free calcium in the cytoplasm of brain neurons in neonatal rats [10]. Water extracts of *P. cocos* have been demonstrated to dose-dependently increase cytosolic free calcium [2] and inhibit glutamate-induced cytosolic free calcium [10] in cells. Similar results have been observed in the primary culture of hippocampal neurons from neonatal rats. Thus, *P. cocos* water extracts can regulate cytoplasmic free calcium in brain nerve cells by affecting a variety of glutamate receptors, such as *N*-methyl-D-aspartate receptor (NMDAR) [10].

The integration of the hippocampus and posterior splenic cortex into a high-level cognitive circuit supports learning and memory in the form of relationships (space, context, and situation) [11]. Impairments in hippocampal neurons might cause abnormal cognitive function in animals [12]. Reduced hippocampal neuronal activities, caused by a reduction in hippocampal neurogenesis or an abnormality of the hippocampal neuronal structure, are always accompanied by cognitive impairments in NMDAR antagonist-treated rats [13, 14]. Schubert et al. [15] reported that active Ras homolog family member A (RhoA) interacts with NMDAR, *α*-amino-3-hydroxy-5-methyl-4-isoxazolepropionic acid receptor, and metabotropic glutamate receptor 1 at the excitatory postsynapsis to maintain NMDAR stabilization and modulate spine actin remodeling [15]. They also mentioned that the activation of RhoA can induce dendritic spine morphology regulation in cultured hippocampal neurons [15]. Cell division cycle 42 (CDC42) levels and dendritic spine density have also been observed to decrease in patients with schizophrenia. A recent report revealed that ketamine can reduce the expression levels of RhoA and Rho-associated coiled-coil containing protein kinase 1 (ROCK1) while simultaneously reducing the proportion of mushroom spines and increasing the proportion of stubby spines in rat hippocampal neurons, which could be involved in the cognitive impairments of schizophrenia [16].

The formation of actin filaments in neuronal cells can induce cytoskeletal rearrangement, which can lead to changes in cell shape and cell function [17]. Cytoskeletal rearrangement also induces synaptic spine elongation, cell migration [18–20], and neuronal plasticity [21, 22], which are important for neuron development and have the same molecular mechanism, namely, the Rho signaling pathway. Furthermore, various studies have mentioned that the calcium concentration in cells plays important roles in regulating Rho signaling regulation and further directional movement, mesodermal sheet migration, cytoskeletal reorganization, and cancer metastasis [18, 23–25]. Our previous study found that APDs might differentially regulate Rho protein expression and activation to further modulate cytoskeletal rearrangement by altering the expression of proteins that are involved in the Rho signaling pathway in B35 and C6 cells and APD-treated rat cortex [26]. We also found that APDs could reverse MK801-induced Rho protein regulation, migration, and actin filament (F-actin) condensation in B35 and C6 cells. In this study, we proposed that *P. cocos* might also provide potential regulatory and modulatory effects on neuronal cell activities by affecting the NMDAR-mediated calcium-related Rho signaling pathway. We aimed to determine whether PRP and WP can regulate the expression and activity of Rho family proteins in B35 and C6 cells. We also examined the effects of PRP and WP on proteins related to Rho signaling. We further investigated whether PRP and WP could enhance the migration ability and F-actin condensation of B35 and C6 cells. Additionally, ketamine-induced B35 cell migration inhibition, which was used to mimic the damaging effect on neuronal cells, was restored by PRP and WP. Ketamine-induced RhoA, CDC42, N-WASP, and ROCK1 expression regulation was also recovered by PRP and WP. We concluded that PRP and WP can modulate the Rho signaling pathway by regulating the expression level and activity of RhoA and CDC42 but not Rac1, thereby increasing cell migration and the F-actin concentration.

## 2. Materials and Methods

### 2.1. Preparation of PRP and WP

Herbal powder extracts of PRP and WP were obtained from Sun Ten Pharmaceutical Company (New Taipei City, Taiwan). To prepare the PRP and WP solutions, herbal powder extracts of PRP and WP were added to sterilized double-distilled H_2_O and left at room temperature for 6 h at a final concentration of 10 mg/ml. The PRP and WP solutions were then centrifuged, and the corresponding supernatants were collected and stored at −20°C until use.

### 2.2. Cell Culture and PRP/WP Treatment

B35 and C6 cells were obtained from the Bioresource Collection and Research Center of the Food Industry Research and Development Institute (Hsinchu City, Taiwan). B35 cells are a rat neuroblastoma cell line used to observe drug effects on neuronal cells. Glial cells can provide nutritional and physiological support, such as neurotransmitter reuptake and recycling to neuronal cells. C6 cells are a glioblastoma cell line used for observing drug effects on glial cells. C6 cells were also used to compare the drug effects between B35 and C6 cells. B35 cells were cultured in modified Eagle's medium (Invitrogen Life Technologies) with fetal bovine serum (10%) (Invitrogen Life Technologies). PRP or WP was added once a day to a final concentration of 10 *μ*g/ml for seven days. To examine the effects of PRP or WP on ketamine-treated B35 cells, B35 cells were treated with ketamine (100 *μ*g/ml) daily for seven days followed by the addition of ketamine (100 *μ*g/ml) and ketamine in combination with PRP (10 *μ*g/ml) or WP (10 *μ*g/ml) daily for another seven days. C6 cells were cultured in high-glucose Dulbecco's modified Eagle's medium (Invitrogen Life Technologies, Carlsbad, CA, USA) containing L-glutamine (2 mm), fetal bovine serum (2%) (Invitrogen Life Technologies), and heat-inactivated horse serum (10%) (Invitrogen Life Technologies). The C6 cells were subcultured when the cell density reached 70% confluence to prevent abnormal S100 protein production. Both cell lines were cultured in a CO_2_ incubator with 5% CO_2_ at 37°C. The cells were harvested for examination after they had been treated with PRP or WP for seven days.

### 2.3. Total Protein Extraction and Western Blot Analysis

B35 and C6 cells were lysed in mammalian protein extraction buffer (GE Healthcare Bio-Sciences, Uppsala, Sweden) to extract total proteins according to the manufacturer's procedures. Protease inhibitor mix (GE Healthcare Bio-Sciences) and phosphatase inhibitors (2 mm NaF and 1 mm Na_3_VO_4_) were added to the lysis buffer to prevent protein degradation. In total, 10–50 *μ*g of the total protein extracts was analyzed using 8%, 10%, or 12.5% sodium dodecyl sulfate-polyacrylamide gel electrophoresis according to the molecular weight of the target proteins. The separated proteins were then transferred from the gels to polyvinylidene difluoride membranes and blocked with membrane blocking solution (Life Technology, Frederick, MD, USA). *β*-Actin was used as an internal calibration control for all of the target proteins. Specific primary antibodies and horseradish peroxidase-conjugated goat anti-mouse or anti-rabbit antibodies (cat. nos. 401215 and 401315; Calbiochem, Darmstadt, Germany) were used to detect the target protein bands. An Amersham ECL kit (Amersham, Bucks, UK) was then used to reveal the protein bands.

### 2.4. RhoA/Rac1/Cdc42 Activity Assay

Rho protein activity was analyzed using the RhoA/Rac1/Cdc42 activity assay kit (Cell Biolab, San Diego, CA, USA). In brief, cells cultured in a 10 cm dish were washed with PBS and lysed in 0.5 mL of lysis buffer containing protease inhibitor mix (GE Healthcare Bio-Sciences) and phosphatase inhibitors (2 mm NaF and 1 mm Na_3_VO_4_). After centrifugation at 14,000 × *g* for 5 min, 300 *μ*g of protein from each cellular lysate was adjusted to a total volume of 1 ml with 1 × assay lysis buffer. Then, each sample received 40 *μ*l of resuspended Rhotekin RBD or PAK PBD agarose beads, and all assay mixtures were incubated for one hour at 4°C with gentle shaking. The beads were then collected by centrifugation at 14,000 × *g* for 10 s followed by removing the supernatants and washing the bead pellets three times with 0.5 ml of 1 × assay lysis buffer. The beads were resuspended in 40 *μ*l of 2 × SDS-PAGE sample buffer and boiled for 5 min. The samples were then analyzed by western blot and detected with anti-RhoA, anti-CDC42, or anti-Rac1 antibodies.

### 2.5. Cell Migration Assay

To examine the migration ability of B35 and C6 cells, they were treated with PRP or WP for seven days followed by seeding 10^4^ PRP- or WP-treated B35 cells and 5 × 10^3^ PRP- or WP-treated C6 cells into a Transwell insert (pore size, 8 *μ*m) (Costar; Corning Incorporation, Kennebunk, ME, USA) set in a 24-well tissue culture plate. The cells were cultured for another 24 h with PRP or WP for continuous stimulation. To examine the effects of PRP or WP on the migration ability of ketamine-treated B35 cells, B35 cells were treated with ketamine for seven days, followed by treatment with ketamine, ketamine with PRP, or ketamine with WP for another seven days. A total of 10^4^ drug-treated B35 cells were seeded into Transwell inserts and cultured for another 24 h with ketamine, PRP, or WP for continuous stimulation. The migrated B35 and C6 cells were washed with 1 × phosphate-buffered saline (PBS) and fixed with methanol and stained with 50 *μ*g/ml propidium iodide solution (Sigma, Saint Louis, MO, USA) for 30 min. The number of cells on the membrane was then counted under a microscope at 40 × magnification. The experiments were performed in triplicate for statistical analysis.

### 2.6. Phalloidin Staining of B35 and C6 Cell F-Actin

Fluorescently labeled phalloidin can stably and specifically bind to F-actin filaments in cells. To observe the F-actin condensation induced by PRP and WP, B35 and C6 cells (5 × 10^3^) treated with PRP or WP for seven days were seeded into a 6-well plate with poly-L-lysine-coated coverslips and then cultured with PRP or WP for another two days accordingly. After the two-day incubation, the coverslips were collected and washed with 1 × PBS. Cells on the coverslips were then fixed in methanol. Subsequently, the cells were washed with 1 × PBS and stained with 1 × phalloidin solution (CytoPainter Phalloidin-iFluor 488 Reagent, ab176753; Abcam, Waltham, MA, USA) at room temperature for 90 min. Residual phalloidin was washed off two to three times with 1 × PBS. The coverslips were mounted and sealed on glass slides and then viewed under a fluorescence microscope.

### 2.7. Statistical Analysis

Student's *t*-tests were performed to analyze differences in the cell migration ability and normalized expression levels of the examined target proteins between the control cells and PRP- or WP-treated B35 and C6 cells. SPSS 20.0 was used for statistical analysis. *p* values of <0.05 (^*∗*^) and <0.01 (^*∗∗*^) were defined as statistically significant.

## 3. Results

### 3.1. PRP and WP Induced Regulation of the Rho Family Proteins

**Figure 1:**
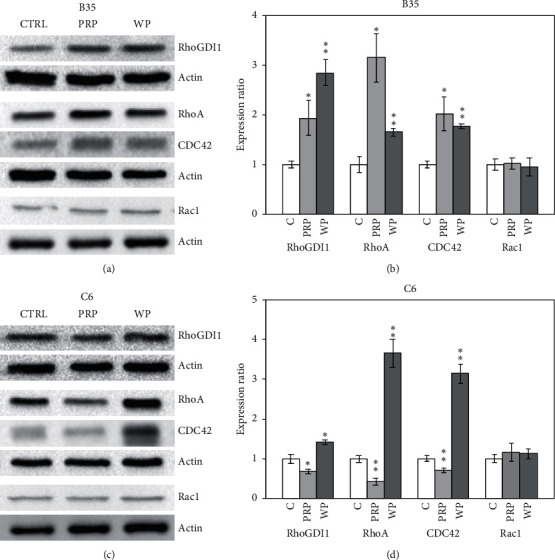
*Poria cocos* regulated RhoGDI1, RhoA, and CDC42 expression in B35 and C6 cells. The expression levels of RhoGDI1, RhoA, CDC42, and Rac1 were examined using western blotting in this experiment. B35 (a, b) and C6 (c, d) cells were treated with PRP (10 *μ*g/ml) or WP (10 *μ*g/ml). The bar charts were calculated based on triplicate western blot data from three batches of PRP- or WP-treated cells using Student's *t*-test (^*∗*^*p* < 0.05; ^*∗∗*^*p* < 0.01). C/CTRL: control; PRP: *Poria cocos* cum Radix Pini; WP: White Poria.

**Figure 2:**
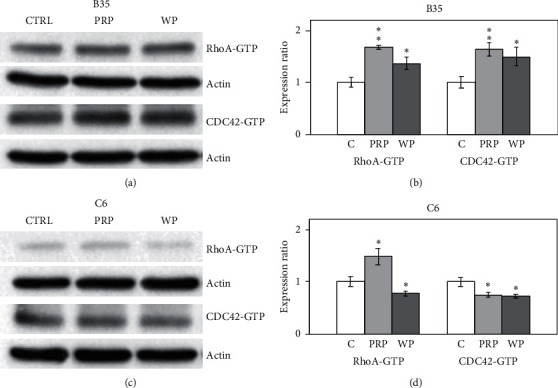
*Poria cocos* induced activation of RhoA and CDC42 in B35 and C6 cells. The expression levels of activated RhoA, CDC42, and Rac1 were examined using western blotting in this experiment. B35 (a, b) and C6 (c, d) cells were treated with PRP (10 *μ*g/ml) or WP (10 *μ*g/ml). The GTP form of Rho protein was pulled down and analyzed. The bar charts were calculated based on triplicate western blot data from three batches of PRP- or WP-treated cells using Student's *t*-test (^*∗*^*p* < 0.05; ^*∗∗*^*p* < 0.01). C/CTRL: control; PRP: *Poria cocos* cum Radix Pini; WP: White Poria.

RhoA, CDC42, and Rac1 are major Rho family proteins that can be regulated by RhoGDP-dissociation inhibitor 1 (RhoGDI1) and affect the modulation of Rho signaling. We found that PRP and WP increased the expression of RhoGDI1 (*p* < 0.05 for PRP and *p* < 0.01 for WP) in B35 cells (Figures 1(a) and 1(b)). Moreover, PRP and WP increased the expression of RhoA (*p* < 0.05 for PRP and *p* < 0.01 for WP) and CDC42 (*p* < 0.05 for PRP and *p* < 0.01 for WP) in B35 cells (Figures 1(a) and 1(b)). Downregulation of RhoGDI1 (*p* < 0.05) was observed in C6 cells treated with PRP (Figures 1(c) and 1(d)). PRP also reduced the expression of RhoA (*p* < 0.01) and CDC42 (*p* < 0.01) in C6 cells (Figures 1(c) and 1(d)). In contrast, WP increased the expression of RhoGDI1 (*p* < 0.05), RhoA (*p* < 0.01), and CDC42 (*p* < 0.01) in C6 cells (Figures 1(c) and 1(d)). Furthermore, neither PRP nor WP affected Rac1 expression in B35 and C6 cells (Figure 1). A pulldown assay was also performed to examine the activation of the Rho family proteins by measuring the expression of GTP form Rho family proteins. We observed that the GTP forms of RhoA (activated RhoA; RhoA-GTP) and CDC42 (activated CDC42; CDC42-GTP) were increased by either PRP (*p* < 0.01) or WP (*p* < 0.05) in B35 cells (Figures 2(a) and 2(b)). In C6 cells, PRP increased RhoA-GTP (*p* < 0.05) and decreased CDC42-GTP (*p* < 0.05) levels, while WP decreased RhoA-GTP (*p* < 0.05) and CDC42-GTP (*p* < 0.05) levels (Figures 2(c) and 2(d)). No GTP form of Rac1 (activated Rac1; Rac1-GTP) was detected in either the B35 or C6 cells in this study.

### 3.2. PRP and WP Modulated F-Actin Condensation and RhoA-Related Rho Signaling

**Figure 3:**
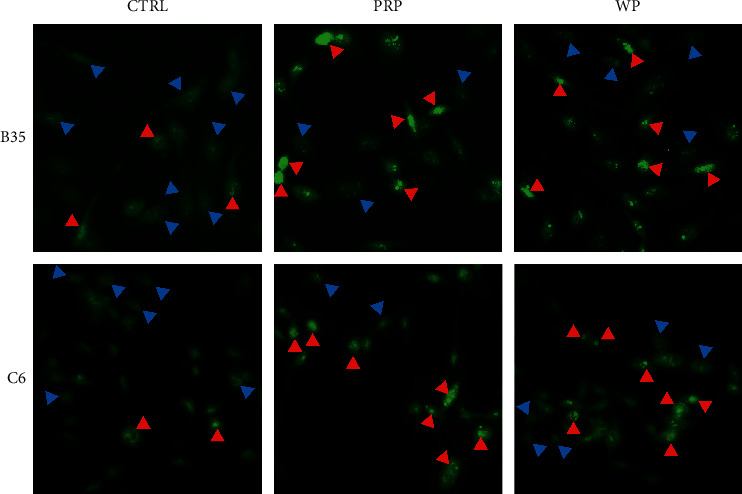
*Poria cocos* induced actin filament condensation in B35 and C6 cells. B35 and C6 cells were treated with PRP (10 *μ*g/ml) or WP (10 *μ*g/ml) for seven days and then stained with CytoPainter Phalloidin-iFluor 488 Reagent. The blue arrows indicate uniformly distributed F-actin, and the red arrows indicate condensed F-actin. C/CTRL: control; PRP: *Poria cocos* cum Radix Pini; WP: White Poria.

**Figure 4:**
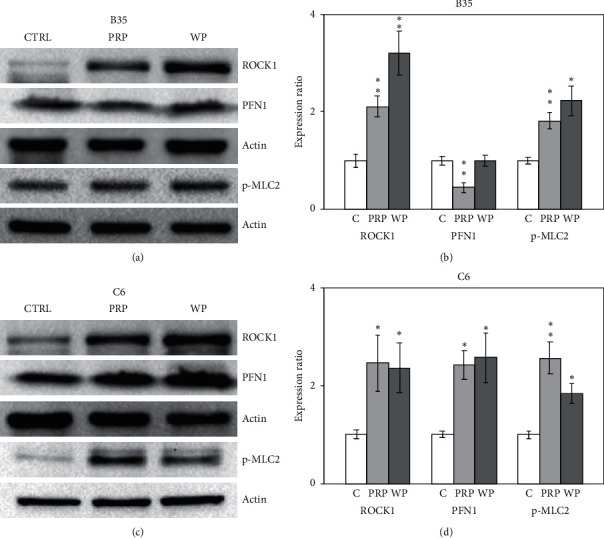
*Poria cocos* induced RhoA-related Rho signaling regulation. The expression levels of ROCK1, N-PFN1, and p-MLC2 were examined using western blotting in this experiment. B35 (a, b) and C6 (c, d) cells were treated with PRP (10 *μ*g/ml) or WP (10 *μ*g/ml). The bar charts were calculated based on triplicate western blot data from three batches of PRP- or WP-treated cells using Student's *t*-test (^*∗*^*p* < 0.05; ^*∗∗*^*p* < 0.01). C/CTRL: control; PRP: *Poria cocos* cum Radix Pini; WP: White Poria.

Rho proteins, including ROCK1, phosphorylated myosin light chain 2 (p-MLC2), and profilin 1 (PFN1), have been proposed to be able to regulate RhoA-related signaling to further modulate F-actin assembly and condensation. Usually, F-actin is evenly distributed in cells (indicated with a blue arrow in Figure 3) and will be condensed (indicated with a red arrow in Figure 3) when regulated. In this study, F-actin condensation in B35 cells was induced by PRP and WP (Figure 3). In addition, PRP and WP also induced F-actin condensation in C6 cells (Figure 3). The expression of ROCK1 was increased by PRP (*p* < 0.01) and WP (*p* < 0.01) in B35 cells (Figures 4(a) and 4(b)). PFN1 expression was reduced by PRP (*p* < 0.05), but not by WP in B35 cells (Figures 4(a) and 4(b)). An increase in the expression of p-MLC2 induced by PRP (*p* < 0.01) and WP (*p* < 0.05) was also observed in B35 cells (Figures 4(a) and 4(b)). Moreover, PRP-induced increased expression of ROCK1 (*p* < 0.05), PFN1 (*p* < 0.05), and p-MLC2 (*p* < 0.01) in C6 cells (Figures 4(c) and 4(d)), whereas WP induced the expression of ROCK1 (*p* < 0.05), PFN1 (*p* < 0.05), and p-MLC2 (*p* < 0.05) in C6 cells (Figures 4(c) and 4(d)).

### 3.3. PRP and WP Modulated CDC42 Migration-Related Rho Signaling

**Figure 5:**
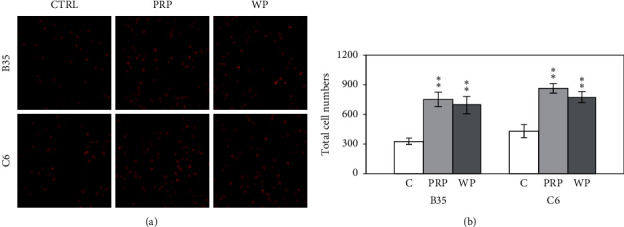
Cell migration regulation induced by PRP and WP in B35 and C6 cells. (a) Propidium iodide staining revealed increased migration of B35 and C6 cells treated with PRP or WP. (b) The bar chart was calculated from three cell migration assays of PRP- or WP-treated cells and analyzed using Student's *t*-test (^*∗*^*p* < 0.05; ^*∗∗*^*p* < 0.01). C/CTRL: control; PRP: *Poria cocos* cum Radix Pini; WP: White Poria.

CDC42 signaling can modulate filopodia and the migration of cells by regulating CDC42 and CDC42 signaling-related proteins, including neuronal Wiskott–Aldrich syndrome protein (N-WASP), p21 (RAC1)-activated kinase 1 (PAK1), and RhoA protein–modulated actin-related protein 2/3 (ARP2/3). Activated CDC42 can modulate cell migration by regulating N-WASP or PAK1. N-WASP can modulate ARP2/3 to further manage actin polymerization and subsequent cell migration. PAK1 can regulate cofilin function to control actin polymerization and cell migration. PRP- or WP-treated B35 and C6 cells were seeded into a Transwell insert, incubated with PRP or WP for another 24 h, and then analyzed to confirm PRP- or WP-induced cell migration. As shown in Figure 5, PRP and WP increased the migration ability of B35 (*p* < 0.01 for both PRP and WP) and C6 (*p* < 0.01 for both PRP and WP) cells.

**Figure 6:**
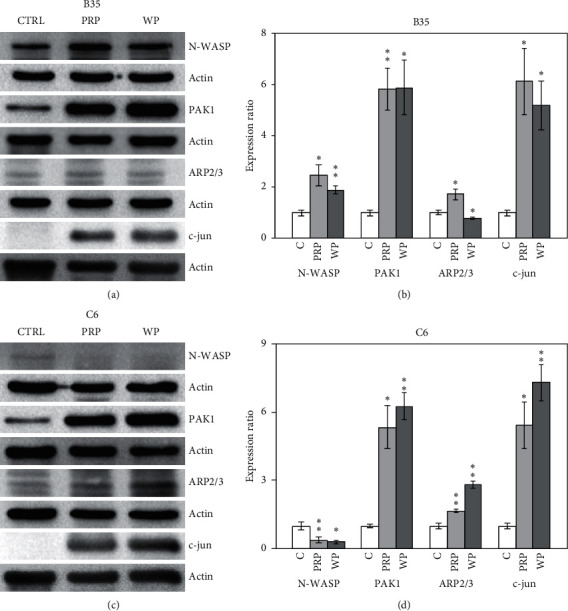
*Poria cocos* induced CDC42/cell migration-related Rho signaling regulation and c-jun expression. The expression levels of N-WASP, PAK1, ARP2/3, and c-jun were examined using western blotting in this experiment. B35 (a, b) and C6 (c, d) cells were treated with PRP (10 *μ*g/ml) or WP (10 *μ*g/ml). The bar charts were calculated based on triplicate western blot data from three batches of PRP- or WP-treated cells using Student's *t*-test (^*∗*^*p* < 0.05; ^*∗∗*^*p* < 0.01). C/CTRL: control; PRP: *Poria cocos* cum Radix Pini; WP: White Poria.

CDC42 signaling-related proteins were also analyzed with western blotting. N-WASP expression in B35 cells was enhanced by PRP (*p* < 0.05) and WP (*p* < 0.01) (Figures 6(a) and 6(b)). Interestingly, PRP and WP reduced N-WASP expression in C6 cells (*p* < 0.01 for PRP and *p* < 0.05 for WP) (Figures 6(c) and 6(d)). The expression of PAK1 was induced by PRP and WP in B35 (*p* < 0.01 for PRP and *p* < 0.05 for WP) (Figures 6(a) and 6(b)) and C6 (*p* < 0.05 for PRP and *p* < 0.01 for WP) cells (Figures 6(c) and 6(d)). ARP2/3 expression was induced by PRP but inhibited by WP in B35 cells. Both PRP (*p* < 0.01) and WP (*p* < 0.01) increased the expression of ARP2/3 in C6 cells (Figures 6(c) and 6(d)).

### 3.4. PRP and WP Modulated c-jun Expression in B35 and C6 Cells

Rho proteins have been reported to regulate c-jun expression and subsequently regulate apoptotic gene expression. As shown in Figures 6(a) and 6(b), increased expression of c-jun was induced by PRP (*p* < 0.05) and WP (*p* < 0.05) in B35 cells. c-jun expression in C6 cells was also increased by PRP (*p* < 0.05) and WP (*p* < 0.01) (Figures 6(c) and 6(d)).

### 3.5. PRP and WP Restored Cell Migration Inhibition and Rho Signaling Regulation in Ketamine-Treated B35 Cells

**Figure 7:**
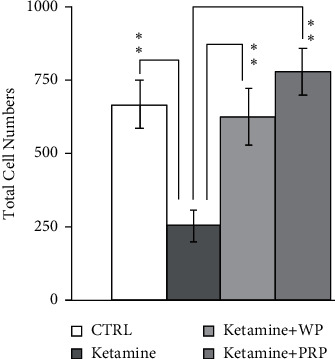
*Poria cocos* reversed ketamine-induced cell migration inhibition in B35 cells. The bar chart was calculated from three batches of cell migration assays. Data were calculated and analyzed by using Student's *t*-test (^*∗*^*p* < 0.05; ^*∗∗*^*p* < 0.01). C/CTRL: control; PRP: *Poria cocos* cum Radix Pini; WP: White Poria.

**Figure 8:**
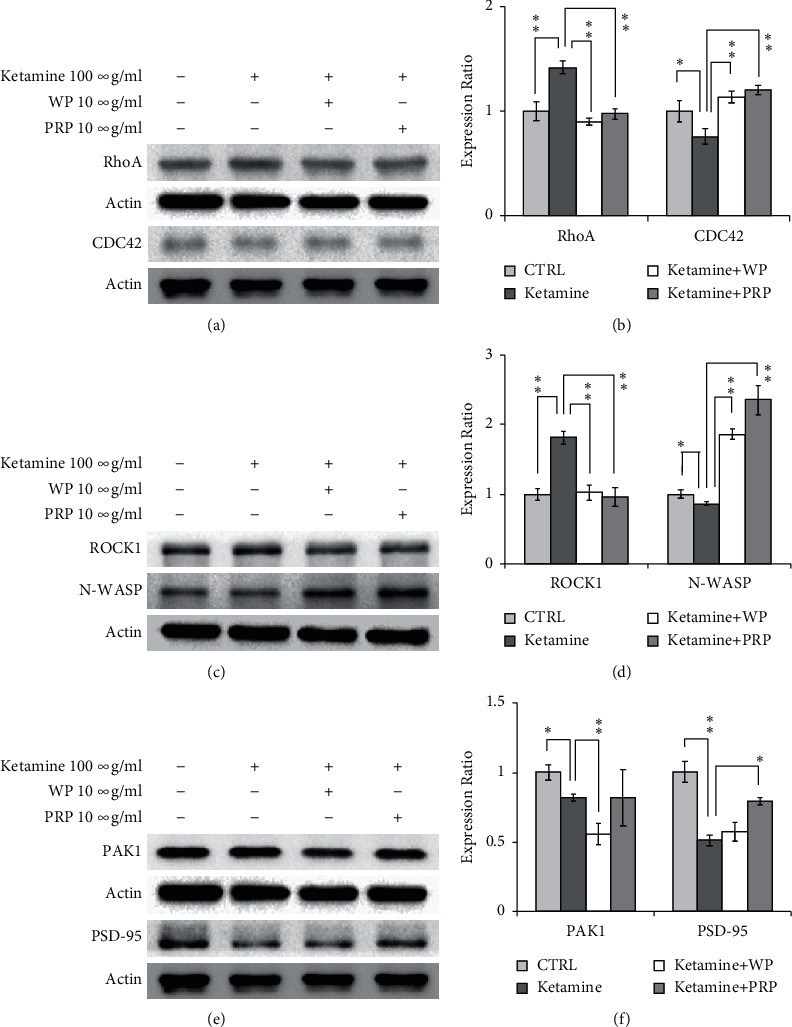
*Poria cocos* reversed ketamine-induced Rho signaling regulation in B35 cells. B35 cells treated with ketamine for seven days were then treated with ketamine or ketamine in combination with PRP or WP for another seven days. The expression levels of RhoA, CDC42, ROCK1, N-WASP, and PAK1 were examined using western blotting in this experiment. The bar charts were calculated based on triplicate western blot data from three batches of drug-treated cells using Student's *t*-test (^*∗*^*p* < 0.05; ^*∗∗*^*p* < 0.01). C/CTRL: control; PRP: *Poria cocos* cum Radix Pini; WP: White Poria.

Ketamine has been reported to induce Rho family protein regulation *in vitro* and *in vivo*. We treated B35 cells with ketamine followed by PRP or WP treatment to further clarify the roles of PRP or WP in Rho signaling-related cytoskeletal regulation in neuronal cells. As Figure 7 shows, B35 cell migration was inhibited by ketamine and was restored by either PRP or WP treatment. We also examined the expression level of some of the Rho signaling proteins in B35 cells treated with ketamine or in combination with PRP/WP. We found that ketamine increased RhoA (*p* < 0.01) and decreased CDC42 (*p* < 0.05) expression in B35 cells, while both PRP and WP reversed ketamine-induced RhoA and CDC42 regulation (Figures 8(a) and 8(b)). Ketamine enhanced Rock1 expression, while PRP (*p* < 0.01)/WP (*p* < 0.01) reversed ketamine-induced ROCK1 induction in B35 cells (Figures 8(c) and 8(d)). We also found that ketamine could induce a reduction in N-WASP expression, which could be restored by PRP or WP treatment (both *p* < 0.01) in B35 cells (Figures 8(c) and 8(d)). Ketamine-reduced PAK1 expression (*p* < 0.05) was further reduced by PRP (*p* < 0.01) but not WP in B35 cells (Figures 8(e) and 8(f)).

Postsynaptic density protein 95 (PSD-95) is a scaffold protein that can promote excitatory synapse maturation of neurons. The expression and accumulation of PSD-95 in postsynaptic dendrites is closely related to Rho protein-dependent cell cytoskeleton regulation [15, 27]. We observed that ketamine decreased PSD-95 expression, which was recovered by WP treatment in B35 cells (Figures 8(e) and 8(f)).

## 4. Discussion

*P. cocos* (Schw.) is a saprophytic fungus that parasitizes various species of *Pinus*, such as *Pinus densiflora* and *Pinus massoniana* [28]. Recent pharmacological research has revealed that *P. cocos* polysaccharides have antinephritic, immunomodulatory, anti-inflammatory, antioxidant, and antitumor effects [29–31]. *P. cocos* has been proposed to treat insomnia through the neurotransmitter *γ*-aminobutyric acid or by stimulating the *γ*-aminobutyric acid AA receptor [32]. *P. cocos* water extracts have also been suggested as herbal medicines for treating depression [33]. A recent study reported that *P. cocos* polysaccharides exhibited neuroprotective effects by regulating various cell functions in an Alzheimer's disease rat model [34]. *P. cocos* has also been found to exhibit antioxidant ability against oxidative stress and to prevent PC12 *β*-amyloid-induced cell death [35].

Rho protein signaling could modulate cell apoptotic gene expression, migration, morphological modulation, and cytoskeletal reorganization by regulating ROCK1 function and downstream MLC2 phosphorylation [16]. The phosphorylation of MLC2 will then induce myosin activation and stress fiber contraction in cells [36, 37]. In addition, the RhoA protein can regulate the expression of ARP2/3 to control cell filopodia and migration [38, 39]. CDC42-induced PAK1/N-WASP activation can also regulate ARP2/3-mediated cytoskeletal remodeling [40]. Recent studies also reported that, in addition to RAC1, PAK1 can be regulated by CDC42 signaling to affect cell migration [41–43]. These findings indicate that neuronal dysfunction caused by abnormal Rho protein expression/activation and downstream Rho protein-mediated pathway signaling may be corrected by reconstructing Rho protein signaling.

In this study, we found that PRP and WP could enhance RhoA, RhoA-GTP, ROCK1, and p-MLC2 expression in B35 neuronal cells. We also observed that PRP and WP could induce CDC42, CDC42-GTP, and N-WASP expression in B35 cells. In addition, PRP and WP enhanced the migration ability and induced F-actin condensation in B35 cells. These results indicate that PRP and WP can regulate cell migration and F-actin aggregation by regulating the Rho signaling pathway, especially by affecting RhoA and CDC42 instead of Rac1.

We also investigated whether ketamine inhibits the migration ability of B35 cells and modulates RhoA, CDC42, ROCK1, and N-WASP expression levels. Ketamine-induced Rho signaling regulation has been linked to various mental disorders. Our results show that PRP and WP can restore ketamine-induced cell migration inhibition and can reverse the ketamine-induced expression regulation of RhoA, CDC42, ROCK1, and N-WASP. These findings suggest that PRP and WP have the potential to correct dysfunction in impaired neurons by regulating abnormal Rho protein signaling.

ROCK1 and PAK1 act as regulators of cofilin activity by modulating LIM kinase activity. High expression levels of ROCK1 and PAK1 can inhibit cofilin activity, leading to further actin polymerization and F-actin stabilization [44, 45]. Our results showed that the expression of ROCK1 and N-WASP induced by PRP/WP enhanced the expression of p-MLC2 and ARP2/3, together with the expression of ROCK1 and PAK1 induced by PRP/WP, leading to cell migration, actin polymerization, and F-actin stabilization. In addition, we found unstable PAK1 expression levels among different control B35 samples and drug-treated B35 cells. We propose that the expression levels of PAK1 might be dynamically controlled by some unrevealed factors. Further studies should be performed to come to a reasonable conclusion about the unstable PAK1 expression.

We also found that WP exhibited effects on Rho protein signaling regulation in C6 cells similar to those observed in B35 cells. WP also induced ROCK1, PAK1, N-WASP, and p-MLC2 expression. These findings suggest that WP could enhance migration and F-actin aggregation in C6 cells by controlling Rho protein expression. Interestingly, the downregulation of RhoA and CDC42 expression induced by PRP could still increase the expression of ROCK1, PAK1, N-WASP, and p-MLC2 in C6 cells and induce cell migration and F-actin concentration.

There may be two reasons for these observations. First, the feedback inhibition of RhoGDI1, RhoA, and CDC42, as well as cross-talk between Rho protein signaling in C6 cells, may be the possible regulatory mechanisms by which cells maintain the signal balance between Rho proteins and related cell functions. Second, the lower expression levels of RhoA and CDC42 in C6 cells may be because there are components in PRP that are different from WP that can specifically reduce the expression levels of RhoA and CDC42 in C6 cells.

## 5. Conclusions

In summary, we found that PRP and WP could enhance migration and F-actin condensation in B35 and C6 cells. We also observed that PRP and WP regulated Rho protein signaling. Ketamine-induced cell migration inhibition and some Rho signaling regulation can be reversed by PRP and WP treatment. These findings suggest that PRP and WP regulate cell migration and F-actin condensation by differentially modulating Rho protein signaling in B35 and C6 cells. These results also show that PRP and WP can regulate Rho protein signaling in neurons and glial cells and, therefore, have the potential to further regulate abnormal cell functions related to mental disorders.

## Data Availability

The datasets used or analyzed in the current study are available from the corresponding author on reasonable request.

## Abbreviations

APD: Antipsychotic drug
ARP2/3: Actin-related protein 2/3
CDC42: Cell division cycle 42
F-actin: Actin filament
NMDAR: N-Methyl-D-aspartate receptor
N-WASP: Neuronal Wiskott–Aldrich Syndrome protein
p-MLC2: Phosphorylated myosin light chain 2
PAK1: p21 (RAC1) activated kinase 1
PFN1: Profilin 1
PRP: *Poria cocos* cum Radix Pini
PSD-95: Postsynaptic density protein 95
RhoA: Ras homolog family member A
RhoGDI1: Rho GDP dissociation inhibitor 1
ROCK1: Rho-associated coiled-coil containing protein kinase 1
WP: White Poria.
